# Low level of plasminogen increases risk for mortality in COVID-19 patients

**DOI:** 10.1038/s41419-021-04070-3

**Published:** 2021-08-05

**Authors:** David Della-Morte, Francesca Pacifici, Camillo Ricordi, Renato Massoud, Valentina Rovella, Stefania Proietti, Mariannina Iozzo, Davide Lauro, Sergio Bernardini, Stefano Bonassi, Nicola Di Daniele

**Affiliations:** 1 Department of Systems Medicine, University of Rome Tor Vergata, Rome, Italy; 2 Department of Neurology, Evelyn F. McKnight Brain Institute, Miller School of Medicine, University of Miami, Miami, FL USA; 3 Department of Human Sciences and Quality of Life Promotion, San Raffaele University, Rome, Italy; 4 Interdisciplinary Center for Advanced Studies on Lab-on-Chip and Organ-on-Chip Applications (ICLOC), University of Rome Tor Vergata, Rome, Italy; 5 Diabetes Research Institute, Cell Transplant Center, Miller School of Medicine, University of Miami, Miami, FL USA; 6 Department of Experimental Medicine, University of Rome Tor Vergata, Rome, Italy; 7 Department of Medical Sciences, Fondazione Policlinico Tor Vergata, Rome, Italy; 8 Clinical and Molecular Epidemiology, IRCCS San Raffaele Roma, Rome, Italy

**Keywords:** Viral infection, Diagnostic markers

## Abstract

The pathophysiology of coronavirus disease 2019 (COVID-19), caused by severe acute respiratory syndrome coronavirus 2 (SARS-CoV-2), and especially of its complications is still not fully understood. In fact, a very high number of patients with COVID-19 die because of thromboembolic causes. A role of plasminogen, as precursor of fibrinolysis, has been hypothesized. In this study, we aimed to investigate the association between plasminogen levels and COVID-19-related outcomes in a population of 55 infected Caucasian patients (mean age: 69.8 ± 14.3, 41.8% female). Low levels of plasminogen were significantly associated with inflammatory markers (CRP, PCT, and IL-6), markers of coagulation (D-dimer, INR, and APTT), and markers of organ dysfunctions (high fasting blood glucose and decrease in the glomerular filtration rate). A multidimensional analysis model, including the correlation of the expression of coagulation with inflammatory parameters, indicated that plasminogen tended to cluster together with IL-6, hence suggesting a common pathway of activation during disease’s complication. Moreover, low levels of plasminogen strongly correlated with mortality in COVID-19 patients even after multiple adjustments for presence of confounding. These data suggest that plasminogen may play a pivotal role in controlling the complex mechanisms beyond the COVID-19 complications, and may be useful both as biomarker for prognosis and for therapeutic target against this extremely aggressive infection.

## Introduction

Since emerging in Wuhan, China, in late 2019, the severe acute respiratory syndrome coronavirus 2 (SARS-CoV-2), the virus that causes coronavirus disease 2019 (COVID-19), unfortunately has spread rapidly worldwide [1–3]. On March 11, 2020, the World Health Organization (WHO) declared COVID-19 a global pandemic (www.who.int) [4, 5]. So far, in all countries of the Western world, we are still fighting against this terrible infective disease even whether vaccines are now available.

The primary mechanism of transmission of SARS-CoV-2 is through infected respiratory droplets occurring by direct or indirect contact with nasal, conjunctival, or oral mucosa. Typical clinical manifestations of COVID-19 include fever, cough, myalgia, headache, and taste and smell dysfunctions [6]. Severe pneumonia as well as acute respiratory distress syndrome (ARDS) are pathological complications of COVID-19 [6]. Regarding the clinical adverse outcomes, including mortality, these are influenced by host factors, such as age, male sex, and comorbidities, which among the most significant include hypertension, diabetes, coronary heart disease, cerebrovascular disease, chronic obstructive pulmonary disease (COPD), and kidney diseases [6]. The immune response seems to have a dual effect: whilst in the initial phases it is pivotal and beneficial [7], in the later advanced stages it may be counterproductive [8], causing lymphopenia [9]. However, also factors directly related to virus, such as viral load kinetics, host immune-system defense mechanisms, and cross-reactive immune memory from previous exposure to other coronaviruses, may influence history of the disease that ranges from absence of symptoms to a mild infection, to a severe disease accompanied by high mortality [10]. While the underlying molecular event is still the focus of intense investigation [3, 11, 12], the monitoring [13, 14] and therapy [15–18] are still waiting innovative effective approach. Nevertheless, COVID-19 worse outcomes are all exacerbated by the SARS-CoV-2 ability to induce vessels thrombosis [19] leading to disseminated intravascular coagulation (DIC) and the cytokines storm [20]. In COVID-19 patients with high rate of mortality, a state of hyperfibrinolysis, characterized by increased fibrin degradation products, such as D-dimer and reduced platelets, is significantly present [21].

Fibrinolysis is a complex process whereby a fibrin-rich thrombus is degraded and remodeled by the protease plasmin, which belongs from the conversion of plasminogen (zymogen) underlying the action of plasminogen activators (tissue-type (tPA) and urokinase-type (uPA)) and plasminogen activator inhibitor-1 (PAI-1) [22]. A role in enhancing virulence and pathogenicity of plasmin(ogen) has been suggested for viruses containing a furin site in their envelope proteins, such as SARS-CoV-2 [23], since plasmin may be able to cleave the S protein of the virus, increasing its ability to bind with angiotensin converting enzyme 2 receptors of host cells, and facilitating virus entry and fusion [23]. Recently, a role for tPA and PAI-1 as biomarkers for COVID-19 has been also proposed since both found were elevated among COVID-19 hospitalized patients [24].

Based on this double role of plasmin(ogen) in facilitating both virus infection and in regulating fibrinolysis, which the deregulation is the most fatal complication of COVID-19, interest it has been focused on this mechanism, even if the association between levels of plasminogen and COVID-19 outcomes was merely speculative and inconclusive [20]. Only a study conducted in 20 patients with COVID-19 and 20 non-COVID-19 sick controls enrolled in the emergency department (ED) reported significant lower plasminogen plasma concentration in hospitalized COVID-19 patients, and in patients with COVID-19 who required intensive care unit (ICU), compared with those were discharged from the ED [25].

Therefore, in the present study, we aimed to analyze the association between plasminogen levels and the main COVID-19-related outcomes, including biological parameters, in a population of infected Caucasian patients.

## Results

### Characteristic of the study population

All characteristics of the study population are reported in Table 1. Results are reported as mean ± standard deviation and in percentage for categorical variables. The mean age of the whole study group was 69.8 ± 14.3 years, and they were mostly men (32, 58%). The overall mortality was 24% and the day of hospitalization 22.8 ± 17.8. As regards comorbidities, 24% had diabetes mellitus, 52% hypertension, 30% cardiovascular disease, 11% stroke, 15% COPD, and 11% were obese. Forty-nine patients (92%) were treated with hydroxychloroquine, 13% with tocilizumab/sarilumab antibodies, and 72% with antivirals.

**Table 1:** Demographic and clinical characteristics of the study population by level of plasminogen.

Variables	Total (*n* = 55)	Plasminogen ≤ 80 (*n* = 19)	Plasminogen > 80 (*n* = 36)	*P* value
Age (years)	69.8 ± 14.3	75.3 ± 11.2	67.3 ± 15.1	0.055
Gender (male, %)	58.2%	66.7%	52.8%	0.331
Smoker (Yes, %)	7.8%	12.5%	5.9%	0.586
Death (Yes, %)	**24.5%**	**55.5%**	**8.3%**	**0.001**
Days of hospitalization	22.8 ± 17.8	22.3 ± 18.7	23.1 ± 17.7	0.884
BMI	24.8 ± 11.55	24.9 ± 11.7	24.7 ± 11.3	0.819
Obesity (Yes, %)	11.3%	17.6%	8.3%	0.593
DM (Yes, %)	24.1%	29.4%	22.2%	0.821
Hypertension (Yes, %)	51.9%	64.7%	44.4%	0.168
CVD (Yes, %)	29.6%	41.2%	25.0%	0.231
Stroke (Yes, %)	11.1%	23.5%	2.8%	0.056
COPD (Yes, %)	14.8%	17.6%	11.1%	0.825
Hydroxychloroquine (Yes, %)	92.5%	88.2%	94.3%	0.831
Tocilizumab/Sarilumab (Yes, %)	13.5%	25%	8.6%	0.253
Antivirals (Yes, %)	72.5%	68.8%	76.5%	0.841
Plasminogen, %	96.3 ± 23.1	66.4 ± 17.0	107.7 ± 12.9	–
Pancreatic amylase	42.65 ± 24.2	39.5 ± 26.8	45.2 ± 22.8	0.618
Total serum alpha amylase	65.3 ± 34.8	58.1 ± 22.8	69.2 ± 40.4	0.304
Lipase	35.2 ± 24.4	37.0 ± 33.8	33.9 ± 18.8	0.575
CRP	95.1 ± 97.0	129.2 ± 87.7	78.4 ± 99.6	0.082
PCT (0.01–0.5)	**0.73** **±** **2.4**	**1.8** **±** **3.9**	**0.2** **±** **0.5**	**0.019**
FBG	**118.9** **±** **59.4**	**142.1** **±** **76.7**	**107.1** **±** **47.0**	**0.047**
HbA1c	48.6 ± 14.4	47.0 ± 22.3	48.6 ± 13.8	0.876
Creatinine	1.14 ± 0.8	1.4 ± 1.1	1.0 ± 0.5	0.065
GFR	74.0 ± 27.1	63.9 ± 32.5	77.3 ± 24.5	0.170
VIT D	13.9 ± 10.1	12.1 ± 8.6	15.2 ± 10.9	0.341
Phosphorus	3.2 ± 0.9	3.4 ± 1.1	3.2 ± 0.7	0.489
Calcium	**8.44** **±** **0.7**	**8.1** **±** **0.6**	**8.6** **±** **0.6**	**0.014**
PTH	144.5 ± 124.8	160.4 ± 143.9	130.4 ± 112.0	0.636
IL-6	**41.9** **±** **55.2**	**61.1** **±** **70.1**	**27.2** **±** **30.7**	**0.017**
TNF-α	27.1 ± 29.8	20.5 ± 12.5	32.1 ± 38.6	0.292
Fibrin	580.7 ± 187.5	586.0 ± 195.6	571.6 ± 184.3	0.792
D-D	1817.5 ± 3808.1	3222.6 ± 6343.7	1146.4 ± 1145.8	0.060
PT/INR	15.6 ± 6.8	17.8 ± 11.2	14.6 ± 2.6	0.106
APTT	32.2 ± 5.3	32.9 ± 6.2	31.7 ± 4.9	0.436

*BMI* body mass index, *DM* diabetes mellitus, *CVD* cardiovascular disease, *COPD* chronic obstructive pulmonary disease, *CRP* C reactive protein, *PCT* procalcitonin, *FBG* fasting blood glucose, *HbA1c* glycated hemoglobin, *GFR* glomerular filtration rate, *VIT D* vitamin D, *PTH* parathyroid hormone, *IL*-6 interlukin-6, *TNF-α* tumor necrosis factor-α, *D-D* D-dimer, *PT/INR* prothrombin time and international normalized ratio, *APTT* activated partial thromboplastin time.

Bold values indicate statistical significance.

In the univariate analysis reported in Table 1, significant association was present between low levels of plasminogen and high rate of mortality (*p* < 0.001), higher levels of pancreatic amylase (*p* < 0.049), higher C reactive protein (CRP) levels (*p* < 0.002), high procalcitonin (PCT) (*p* < 0.019), and high fasting blood glucose (FBG) (*p* < 0.047). Moreover, significant associations were present for lower level of plasminogen and lower level of calcium (*p* < 0.014), higher levels of interleukin-6 (IL-6) (*p* < 0.017), and high levels of D-dimer (*p* < 0.011).

### Low plasminogen levels are associated with higher inflammatory parameters in COVID-19 patients

**Fig. 1:**
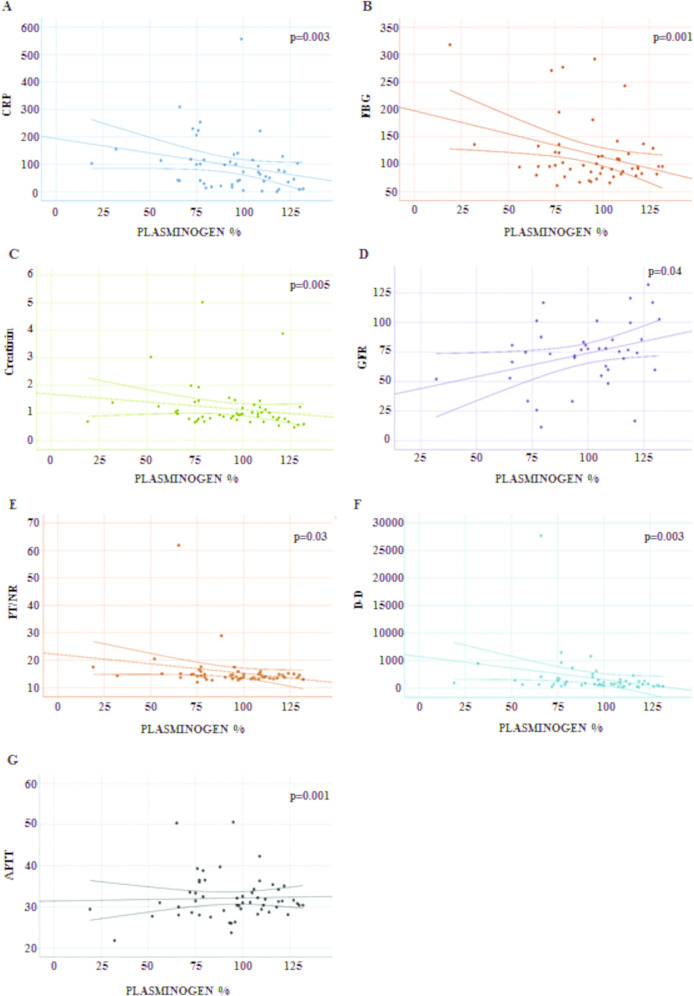
Analysis of the correlation between level of plasminogen and markers related to COVID-9 pathogenesis. Levels of plasminogen were correlated with several markers linked with COVID-19 pathogenesis such as CRP (**A**), FBG (**B**), creatinine (**C**), GFR (**D**), PT/INR (**E**), D-D (**F**), and APTT (**G**). CRP C reactive protein, FBG fasting blood glucose, GFR glomerular filtration rate, PT/INR prothrombin time and international normalized ratio, D-D D-dimer, APTT activated partial thromboplastin time.

Based on the rationale previously reported, and on the results shown in Table 1, we investigated further the association between plasminogen levels and the levels of the most relevant parameters associated to COVID-19 pathogenesis. As reported in the scatterplots in Fig. 1, lower levels of plasminogen resulted in a significant increase of CRP (*r*^2^ = −0.17, *p* = 0.003, Fig. 1A), FBG (*r*^2^ = −0.10, *p* = 0.001, Fig. 1B), and creatinine (*r*^2^ = −0.03, *p* = 0.005, Fig. 1C), a decrease in the glomerular filtration rate (GFR) (*r*^2^ = −0.03, *p* = 0.04, Fig. 1D), and a variation in coagulation parameters, including increase in INR (*r*^2^ = −0.06, *p* = 0.03, Fig. 1E) and in D-dimer (*r*^2^ = −0.06, *p* = 0.003, Fig. 1F), and significant decrease in activated partial thromboplastin time (APTT) (*r*^2^ = −0.001, *p* = 0.03, Fig. 1G). Even the association between plasminogen and levels of PCT, calcium, and IL-6 was inversely related, i.e., *r*^2^ = −0.405, *p* = 0.003; *r*^2^ = −0.333, *p* = 0.014; and *r*^2^ = −0.366, *p* = 0.006, respectively.

**Fig. 2:**
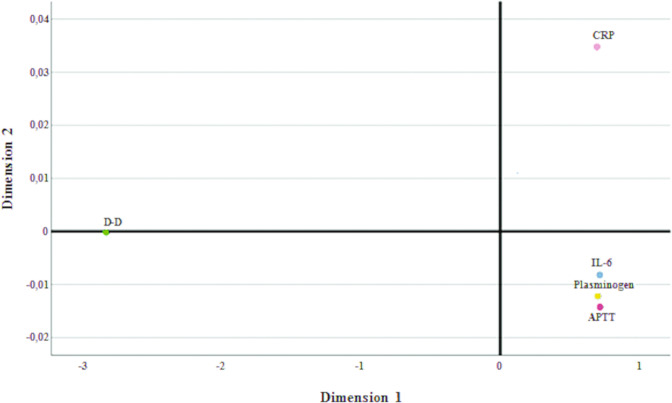
Multidimensional scaling analysis reporting the correlation of the expression of coagulation and inflammatory parameters with plasminogen. IL-6 and APTT tended to cluster together with plasminogen compared to D-dimer and CRP, which tended to be dissociated, and therefore to possibly act independently on different pathways of proinflammation. D-D D-dimer, CRP C reactive protein, IL-6 interlukin-6, APTT activated partial thromboplastin time.

**Fig. 3:**
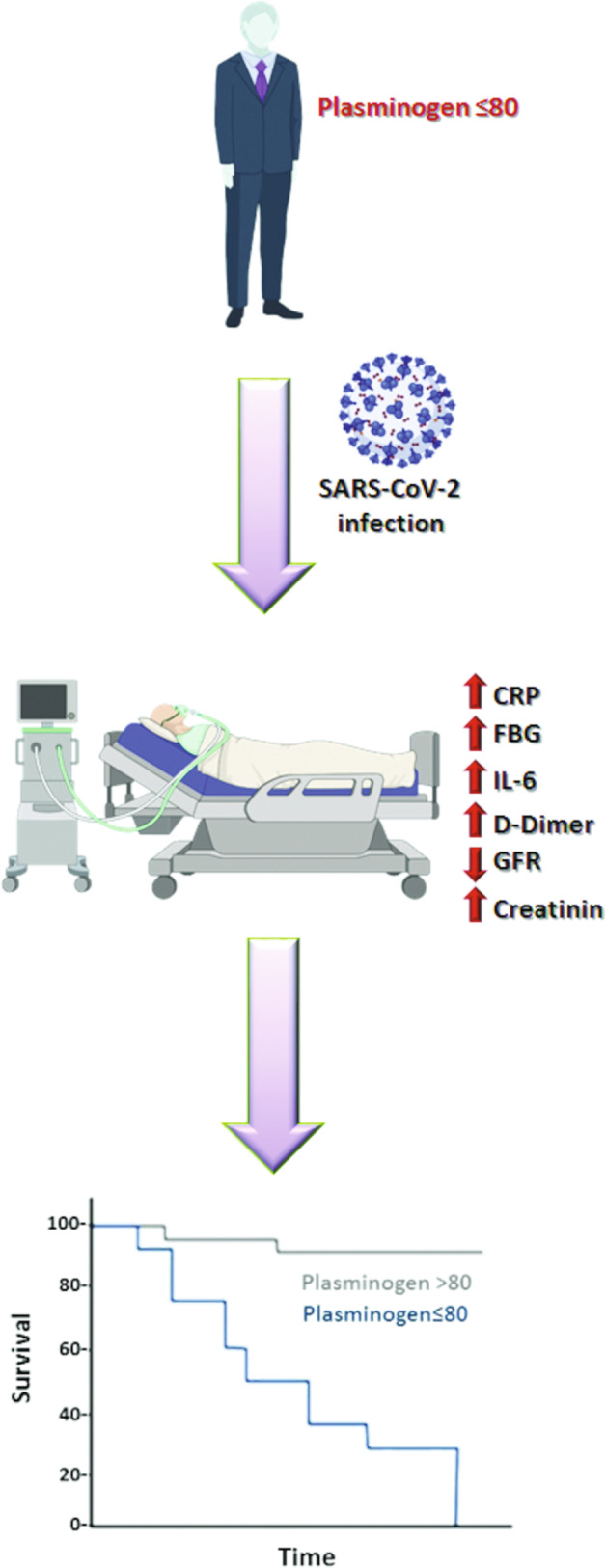
Schematic representation of COVID-19 impact on subjects with lower levels of plasminogen. Subjects with plasminogen ≤ 80 showed poor prognosis following SARS-CoV-2 infection due to alteration in several inflammatory and coagulation-related parameters. CRP C reactive protein, FBG fasting blood glucose, IL-6 interlukin-6, GFR glomerular filtration rate. Created by BioRender.com.

To evaluate the relationships between different inflammatory parameters and to take into account the novel findings that COVID-19-related inflammatory circuits formed by cytokines storm are mediated by an interaction between IL-6 and plasminogen mediators [26], we designed a descriptive multidimensional model including the correlation of the expression of coagulation and inflammatory parameters (Figs. 2 and 3). It was clear as plasminogen, IL-6, and APTT tended to cluster together compared to D-dimer and CRP, which tended to be dissociated, and therefore to possibly act independently on different pathways of proinflammation and DIC.

Taken together, these data suggest a potential relationship between levels of inflammation, mediated by IL-6, levels of circulating plasminogen, as marker of fibrinolysis, and the shorter time in forming clots, as per APTT levels, typical of COVID-19 patients.

### Low level of plasminogen is one of the most significant predictors of mortality in patients with COVID-19

Since the mortality in COVID-19 patients increases in logarithmic way and comorbidities and older age have a great impact on it [27], we aimed to evaluate whether factors, such as alteration in plasminogen levels, that generate the “blood clots storm” may be predictive markers.

Then, by using univariate logistic regression analysis, we tested the most significant parameters found associated with levels of plasminogen in univariate analysis (Table 2). COVID-19 patients with low levels of plasminogen showed a 12-fold significant increase in mortality compared to COVID-19 patients with normal or high levels of plasminogen (OR 12.57, 95% CI 2.46–64.0, *β* = 2.53, *p* = 0.002). By using the median values as cutoff, we also found that in patients with COVID-19, high level of CRP, as marker of inflammation, was associated with up to sevenfold risk of mortality (OR 7.353, 95% CI 1.29–41.7, *β* = 1.99, *p* = 0.02); high FBG, as marker of metabolic dysfunction, was greatly associated until up to 17-fold risk of mortality (OR 17.153, 95% CI 1.72–170.4, *β* = 2.84, *p* = 0.01); high creatinine, as marker of kidney failure, tended to be associated with up to fourfold risk of mortality (OR 4.707, 95% CI 0.95–23.1, *β* = 1.54, *p* = 0.05); and D-dimer, as marker of procoagulation, was associated up to sevenfold risk of mortality (OR 7.655, 95% CI 1.32–44.2, *β* = 2.03, *p* = 0.02) (Table 2).

**Table 2:** Risk of death in COVID-19 patients by level of selected inflammatory parameters (univariate logistic regression analysis).

Variables	*B* (SE)	*P* value	Odds ratio	95% CI
Plasminogen				
>80%	–		1.00	
≤80%	2.531 (0.830)	0.002	**12.57**	2.47–64.00
CRP				
≤72.5	–		1.00	
>72.5	1.995 (0.887)	0.024	**7.35**	1.29–41.79
FBG				
≤96.0	–		1.00	
>96.0	2.842 (1.172)	0.015	**17.15**	1.73–170.45
Creatinine				
≤0.93	–		1.00	
>0.93	1.549 (0.813)	0.057	4.71	0.96–23.15
D-D				
≤812.0	–		1.00	
>812.0	2.035 (0.895)	0.023	**7.66**	1.32–44.27

Analyses based on 52 subjects (13 deaths).

*CRP* C reactive protein, *FBG* fasting blood glucose, *D-D* D-dimer.

Bold values indicate statistical significance.

The relative contribution to the risk of death for all parameters investigated was evaluated with a multiple logistic regression, in a model adjusted also for presence of comorbidities, age, and sex. As reported in Table 3, males and COVID-19 patients older than 70 years of age showed a borderline increase of the mortality risk compared to female and younger patients. In the univariate analysis, similar increases were found in patients with high CRP and D-dimer levels, while after multiple adjustments, the only parameter that remained significantly associated to mortality, with essentially the same risk, was the plasminogen (OR 12.68, 95% CI 1.69–95.0, *β* = 2.02, *p* = 0.01) (Table 3). Taken together these results suggest that in the complex pathway of COVID-19, levels of plasminogen may be indicated as independent predictor of mortality in these patients.

**Table 3:** Risk of death in COVID-19 patients (multiple logistic regression analysis).

Variables	*B* (SE)	*P* value	Odds ratio	95% CI
Plasminogen				
>80%			1.00	
≤80%	2.540 (1.028)	0.013	**12.68**	1.69–95.06
Sex				
Female			1.00	
Male	3.241 (1.745)	0.063	25.56	0.84–160.72
Age (years)				
<70			1.00	
>70	2.023 (1.560)	0.195	7.56	0.36–160.7
D-D				
≤812.0			1.00	
>812.0	1.583 (1.068)	0.138	4.87	0.60–39.50

Analyses based on 52 subjects (13 deaths).

*D-D* D-dimer.

Bold values indicate statistical significance.

## Discussion

In the present study, in an aged population of patients with diagnosis of COVID-19 and with high rate of comorbidity, we demonstrated that low levels of plasminogen were significantly associated with several prognostic parameters of complications, such as inflammatory markers (CRP, PCT, and IL-6), markers of coagulation (D-dimer, INR, and APTT), and markers of organ dysfunctions (FBG and GFR). In the present population, by using a multidimensional model, we showed the tendency of lower levels of plasminogen to cluster with IL-6 and APTT, compared to other parameters of inflammation and coagulation, suggesting a possible pathway that links these three factors in triggering the disseminate intravasal coagulation, as complication of COVID-19. In addition, low levels of plasminogen were associated with 12-fold higher risk for mortality even after multiple adjustments, suggesting its power as independent predictor of worse outcomes in subjects with COVID-19.

Since the beginning it was clear that COVID-19 was a particular viral infection characterized by various complications, often fatal. One of the most important complication, leading to death, as we previously highlighted, is the ability of SARS-CoV-2 to induce disseminated procoagulations [20]. The mechanisms beyond this pathological process are not understood yet, and even in the era of vaccination, scientific committees have an open discussion worldwide on how to prevent and manage this virus-related side effect. Therefore, biomarkers that might predict this complication would be invaluable, especially in triaging patients on hospital admission and in frail and elderly subjects receiving vaccination.

Recently, among blood biomarkers measured from 187 patients admitted at hospital with diagnosis of COVID-19, also including IL-6, Krebs von den Lungen 6, troponin, ferritin, lactate dehydrogenase, B-type natriuretic peptide, PCT, and CRP, the strongest association with negative outcomes, defined as noninvasive ventilation or ICU admission, was the high levels of soluble urokinase plasminogen activator receptor (suPAR) and IL-6 [28]. These results align with our findings since high levels of suPAR lead to lower levels of circulating plasminogen for its degradation [29]. Moreover, in the present study, we found how plasminogen and IL-6 cluster together, along with APTT, in a multidimensional analysis, suggesting that cytokines storm, mainly mediated by IL-6, activates the process of fibrinolysis and the intrinsic and common pathways of the coagulation cascade. This link was already suggested in 91 patients with ARDS where among the proinflammatory cytokines, IL-6 was the most clinically suitable biomarker associated with high levels of PAI-1, and this pathway was beyond the cytokines storm activation in endothelial cells [26].

The role of levels of plasminogen activator and inhibitor in the association with severity of COVID-19 progression has been already reported [30]. However, here, we demonstrated the direct association between the consumption of plasminogen and increased markers of inflammation, coagulation, and organ dysfunctions. To the best of our knowledge, we are the first demonstrating the direct impact of plasminogen levels with COVID-19 outcomes and prognostic parameters. A previous study, in line with our data, demonstrated that lower levels of plasminogen resulted in highest rate of admission to ICU in COVID-19 patients but without reporting the association with other biomarkers of the disease [25].

This association may be explained since the shift in the procoagulant pathways has been observed in the later stages of COVID-19 and the switch of plasmin(ogen) can promote further infection, other than disseminate intravasal coagulation, by cleaving proteins that allow cell infection [31]. These would further justify the association we found in the present study between low levels of plasminogen and high rate of mortality. In univariate logistic regression analysis, we found low levels of plasminogen increased risk of death up to 12-fold, along with other prognostic markers, such as CRP, high FBG, high creatinine, and high D-dimer. These findings, excluding for plasminogen, further confirmed evidence from other studies [32]. However, after multiple logistic regression, which included in the model all comorbidities, the only parameter that maintained significance with the same level of strength was plasminogen. In agreement with our results, a previous study demonstrated as high PAI-1 tracked most closely with impaired oxygenation efficiency in COVID-19 patients, and tPA was the best predictor of death, as activator of plasminogen, then leading to its consume [24]. These data are of particular importance since understanding the mechanisms beyond the COVID-19 complications, especially those linked with the disseminate vascular coagulation that kills these patients, would be helpful both as biomarker of disease to prevent mortality and also to promote or adjust therapies modulating fibrinolysis, like the trial already running with alteplase (https://clinicaltrials.gov/ct2/show/NCT04640194?term=tpa&cond=Covid19&draw=2&rank=6).

We need to acknowledge some limitations for the present study over the current search for the underlying molecular mechanism of the disease [33–35]. Considering the emerging role of predictive medicine [36–38] and personalized therapy [39–42], this will soon become essential. Currently, we are far from this goal, as, for example, we do not know the role of Bcl-2 family [43, 44], degradation pathways [45, 46], autophagy [47–51], and, as described in this paper, plasminogen. First, the number of subjects enrolled in the study was small due to the research restrictions during the pandemic. Then, serial plasminogen measurements at different time-points will be also necessary in future studies to delineate this potential mechanism.

We would like to highlight that atomization inhalation of 10 mg plasminogen dissolved in 2-ml sterile water given twice daily in five clinically moderate patients with COVID-19 has been shown to improve the conditions of lung lesions, heart rate, and oxygen saturation, about 1 h after the first inhalation, suggesting that plasminogen may be effective and efficient in treating several complications during COVID-19 infections. The levels of plasminogen were not measured in the patients [52]. In this study, we did report a strong association between low levels of plasminogen and high rate of mortality in patients with COVID-19. Further studies are imperative to better understand this mechanism and to provide a timely identification of patients at higher risk of complications.

## Material and methods

### Study population

Fifty-five SARS-CoV-2-positive adults were enrolled in an open study by the Internal Medicine Unit, Departments of Systems Medicine and Experimental Medicine, University of Rome Tor Vergata from May to September 2020. The diagnosis was made according to the relevant diagnostic or classification criteria, and COVID-19 severity was classified according to WHO guidance (https://apps.who.int/iris/handle/10665/330893). Only patients with SARS-CoV-2 confirmed by polymerase chain reaction from nasopharyngeal swabs were included in the study. All demographic and clinical data were collected from medical charts and are reported in Table 1. The ethical committee of Tor Vergata University/Hospital approved this study (protocol no. 48.20, version 2020). Informed written consent was obtained from each patient. The study was done in accordance with the ethical principles of the Declaration of Helsinki and the Guidelines for Good Clinical Practice.

### Blood parameters and plasminogen measurement

Blood samples were collected via routine blood draws for clinical indications at Emergency Department and Internal Medicine Unit of University of Rome Tor Vergata at the time of admission to Internal Medicine Unit, according to standard procedure for cellular [53–55] and clinical biochemistry [56] or redox analysis [56–60], as previously described [61, 62]. Plasminogen blood levels were measured at the same time. Within 3 h of collection, samples were centrifuged at 2000 × *g* for 15 min at 4 °C and subsequently frozen at −80 °C until analysis. Circulating (plasma) plasminogen activity was measured by performing an automated chromogenic assay (HemosIL, Werfen, Italy) and analyzed by using a Behring Nephelometer II System (Siemens Medical Solutions, Inc, Malvern, PA, USA) according to the manufacturer’s protocol and literature [63]. Based on the plasminogen kit protocols and reference range [63], we classified low level as the patients with values of plasminogen ≤ 80 (*n* = 19), and normal/high as those with values > 80 (*n* = 36).

### Statistical analysis

Main characteristics of the study group were explored using descriptive statistics. Patients with levels of plasminogen below 80% were compared with those over this threshold, using Student’s *t* test and χ^2^ test for continuous and categorical variables, respectively. Bidimensional descriptive graphics were based on the Pearson’s *r* correlation coefficient, while the multidimensional plot was generated using the procedure Multidimensional Scaling Model by SPSS (IBM SPSS Statistics for Windows, Version 26.0), a procedure which helps understanding data clustering assigning observations to specific locations in a conceptual space. To provide a quantitative estimate of the risk of death associated to those variables, which from descriptive analyses resulted more associated with inflammatory parameters, a univariate logistic regression model was run. Confounding effect was taken subsequently into account including in the logistic model, an appropriate set of actual and potential confounders related to individual characteristics, lifestyle, and dietary patterns. For each model, the occurrence of overdispersion was checked by comparing the residual deviance with its degrees of freedom. The likelihood ratio test was applied to assess the significance of each variable in the logistic model. STATA (Stata Statistical Software: Release 11, StataCorp LP) software was used for modeling.

## Data Availability

All data generated or analyzed during this study are included in this article.
